# Improving the population genetics toolbox for the study of the African malaria vector *Anopheles nili*: microsatellite mapping to chromosomes

**DOI:** 10.1186/1756-3305-4-202

**Published:** 2011-10-19

**Authors:** Ashley Peery, Maria V Sharakhova, Christophe Antonio-Nkondjio, Cyrille Ndo, Mylene Weill, Frederic Simard, Igor V Sharakhov

**Affiliations:** 1 Department of Entomology, Virginia Polytechnic and State University, West Campus Drive, Blacksburg, VA 24061, USA; 2 Malaria Research Laboratory, Organisation de Coordination pour la lutte contre les Endémies en Afrique Centrale (OCEAC), Yaounde, BP 288, Cameroon; 3 Institut des Sciences de l'Evolution, Université Montpellier 2, Centre National de la Recherche Scientifique, Place Eugène Bataillon, C.C. 065, 34095 Montpellier, France; 4 Unité Mixte de Recherche 224 'Maladies Infectieuses et Vecteurs: Ecologie, Genetique, Evolution et Controle (MIVEGEC)', Team 'Biology, Ecology and Evolution of vector Systems (BEES)', Institut de Recherche pour le Developpement (IRD), BP 64501, Montpellier 34394, France; 5 Institut de Recherche en Sciences de la Santé (IRSS), 399 Avenue de la Liberte, Bobo Dioulasso, 01BP171, Burkina Faso

**Keywords:** Chromosome inversions, genome sequence, malaria vector, microsatellite markers, population structure

## Abstract

**Background:**

*Anopheles nili *is a major vector of malaria in the humid savannas and forested areas of sub-Saharan Africa. Understanding the population genetic structure and evolutionary dynamics of this species is important for the development of an adequate and targeted malaria control strategy in Africa. Chromosomal inversions and microsatellite markers are commonly used for studying the population structure of malaria mosquitoes. Physical mapping of these markers onto the chromosomes further improves the toolbox, and allows inference on the demographic and evolutionary history of the target species.

**Results:**

Availability of polytene chromosomes allowed us to develop a map of microsatellite markers and to study polymorphism of chromosomal inversions. Nine microsatellite markers were mapped to unique locations on all five chromosomal arms of *An. nili *using fluorescent *in situ *hybridization (FISH). Probes were obtained from 300-483 bp-long inserts of plasmid clones and from 506-559 bp-long fragments amplified with primers designed using the *An. nili *genome assembly generated on an Illumina platform. Two additional loci were assigned to specific chromosome arms of *An. nili *based on *in silico *sequence similarity and chromosome synteny with *Anopheles gambiae*. Three microsatellites were mapped inside or in the vicinity of the polymorphic chromosomal inversions *2Rb *and *2Rc*. A statistically significant departure from Hardy-Weinberg equilibrium, due to a deficit in heterozygotes at the *2Rb *inversion, and highly significant linkage disequilibrium between the two inversions, were detected in natural *An. nili *populations collected from Burkina Faso.

**Conclusions:**

Our study demonstrated that next-generation sequencing can be used to improve FISH for microsatellite mapping in species with no reference genome sequence. Physical mapping of microsatellite markers in *An. nili *showed that their cytological locations spanned the entire five-arm complement, allowing genome-wide inferences. The knowledge about polymorphic inversions and chromosomal locations of microsatellite markers has been useful for explaining differences in genetic variability across loci and significant differentiation observed among natural populations of *An. nili*.

## Background

*Anopheles gambiae*, *An. arabiensis*, *An. funestus*, and *An. nili *are the major malaria vectors in sub-Saharan Africa because they are anthropophilic and susceptible to *Plasmodium falciparum *[1-3]. These species belong to species complexes or groups, and members within these complexes/groups vary significantly in their vectorial capacity. Moreover, species can be further sub-divided into populations adapted to different environments. Some malaria control initiatives have failed because they targeted the wrong species or population [4,5]. Understanding and targeting the heterogeneity and complexity of all major vector species and populations is necessary for effective vector control and malaria eradication [6].

Most studies of African malaria vectors have involved only *An. gambiae*, *An. arabiensis*, and *An. funestus*, while research on other important malaria vectors has critically lagged behind. For *An. nili*, this is partly because molecular and cytogenetic tools for characterizing population structure, ecological adaptation, and taxonomic status have been lacking. *Anopheles nili *is widely distributed and contributes substantially to malaria transmission in the African savannah and forested areas, where it breeds in lotic streams and rivers [7,8]. Sporozoite rates in this species can reach 3%, and the annual entomological inoculation rates can be over 100 [9]. For example, *An. nili *is highly anthropophagous and responsible for 10.2% of malaria transmission in the densely populated area surrounding Yaounde, the capital of Cameroon [10]. Gaps in our knowledge of this vector represent a critical barrier to progress in the field of vector biology. Recent findings of circulation of *P. falciparum *and other *Plasmodium *species in great apes and other primates [11-13] raise concerns about pathogen transfer between humans and primates, and highlight the need to improve our knowledge of malaria vectors that inhabit forested areas in Central Africa.

Multi-allelic microsatellites are informative markers for inferring the population and taxonomic status of disease vectors and parasites [1,14-26]. Microsatellites are hyper-variable markers that tend to evolve neutrally. Eleven polymorphic microsatellite markers have been developed for *An. nili *[27]. Recently, the level of genetic variability and differentiation has been explored among nine populations of *An. nili *from Senegal, Ivory Coast, Burkina Faso, Nigeria, Cameroon, and The Democratic Republic of Congo (DRC) [1]. Genetic variability was determined by assessing polymorphisms at these 11 microsatellite markers, together with sequence variations in four genes within the ITS2, 28S rDNA subunit D3, and mitochondrial DNA. High *F*_*ST*_ estimates based on microsatellites (*F*_*ST*_ > 0.118, *P *< 0.001) were observed in all comparisons between Kenge in the DRC, and all other populations sampled from Senegal to Cameroon. Sequence variation in mtDNA genes matched these results; however, low polymorphism in rDNA genes prevented detection of any population substructure at this geographical scale. Both local adaptation and geographic isolation could cause this differentiation. Geographic isolation should affect all markers, even if they are unlinked (i.e. located in different chromosomes). However, chromosomal locations of the microsatellite markers and, therefore, the degree of their physical independence in the genome were unknown. Furthermore, because reduced recombination and increased selection within or near polymorphic inversions can result in estimates of gene flow that may differ significantly from those based on loci elsewhere in the genome [28,29], it would also be important to know the location of microsatellite markers with respect to polymorphic inversions in *An. nili *when performing population genetic analyses.

Polymorphic chromosomal inversions are usually under selection and, thus, are useful markers for studying ecological adaptations of malaria mosquitoes [30-32]. The polymorphic inversions of chromosome 2 of *An. gambiae *have been associated with the arid Sahel Savanna [33-37] and with tolerance to desiccation and heat [38,39]. Moreover, frequencies of these inversions are higher indoors where the nocturnal saturation deficit is higher than outdoors [35]. Such ecological heterogeneity has important consequences for vector control. For example, indoor residual spraying of insecticides affected only indoor populations of *An. gambiae *in the Garki malaria control project in Nigeria [40]. Our previous cytogenetic analysis demonstrated that two polymorphic inversions, *2Rb *and *2Rc*, are present simultaneously in an *An. nili *mosquito. However, they display very different patterns of polymorphism. Frequencies of inverted and standard *2Rb *variants were almost equal (with a deficiency of heterozygotes) in Burkina Faso, whereas only the standard arrangement was found in Cameroon. In contrast, inversion *2Rc *occurred at higher frequency (without a deficiency of heterozygotes) in the dry savannah of Burkina Faso (83%) and at lower frequency in the humid rainforest of Cameroon (0.6%) [32]. Moreover, inversion *2Rc *was found in the mountainous area (Magba), but not in the forested area (Mbebe) of Cameroon. These observations suggest the involvement of inversions in local adaptation (*2Rb*) or in an ecogeographic adaptive cline from dry to more humid environments (*2Rc*). Because *An. nili *is a forest-savannah transition species, polymorphic inversions could provide genetic plasticity that allows this species to expand its range from dry savannah to deforested areas of Central Africa, where most of the human population is present. The relationship between these two inversions has not been studied. For example, it would be useful to know if inversions *2Rb *and *2Rc *are in linkage disequilibrium (LD) in natural populations of *An. nili*.

In this study, we mapped nine microsatellite markers to polytene chromosomes of *An. nili *using fluorescent *in situ *hybridization (FISH). Plasmid clones of the *An. nili *microsatellites and/or *ad hoc *DNA fragments amplified from a low coverage assembly of the *An. nili *genome were used as probes. The microsatellites hybridized to unique locations on all chromosomes both inside and outside polymorphic inversions. We further demonstrated highly significant linkage disequilibrium between inversions *2Rb *and *2Rc*. This knowledge about polymorphic inversions and chromosomal locations of microsatellite loci helped us to better understand genetic variations and differentiation in natural populations of *An. nili*.

## Results

### Experimental approaches to microsatellite mapping

In the current study, we used three experimental approaches to map microsatellite markers to the polytene chromosomes from ovarian nurse cells of wild female *An. nili *specimens collected in Burkina Faso. In the first approach, microsatellites were amplified from genomic DNA using specific primers, which were previously developed [27]. All microsatellites were successfully amplified from the genomic DNA. However, because of the small size of the products (approximately 90-230 bp), a majority of the probes failed to hybridize to chromosomes. Only one microsatellite, 1F43, was mapped by this method. In the second approach, inserts containing microsatellites previously cloned in the pUC18 plasmid [27] were amplified using M13 forward and reverse primers. The insert sizes in this case ranged from 300 to 483 bp. Most of the microsatellites, except F41, B115, 2C157, and A154 were successfully labeled and hybridized to polytene chromosomes. Marker 1F43 was also mapped by the second approach to the same chromosomal region as in the first approach. In the third approach, we used a recently obtained genomic sequence assembly of *An. nili *to identify the microsatellite loci via BLASTN search and to design primers for PCR. These primers allowed the amplification of 506-559 bp-long PCR products containing the microsatellites that could not be hybridized previously. The *An. nili *genome was sequenced by Illumina 72 bp paired-end method using genomic DNA isolated from two individual larvae collected in Dinderesso, Burkina Faso. The assembly consisted of 51,048 contigs with a total length of 98,320,874 bp. The average contig length was 1,926 bp and the maximum contig length was 30,512 bp. Primers were designed for microsatellites B115, 2C157, and A154 (accession numbers: JF742787, JF742788, JF742789) based on sequences identified by BLASTN (Table 1). We successfully mapped microsatellites B115 and 2C157 to polytene chromosomes using this approach. However, microsatellite A154 failed to hybridize to chromosomes despite several attempts. The BLASTN search yielded multiple hits for microsatellite locus F41 in the *An. nili *genome because of widespread occurrence of the (CT)_11_TT(CT)_8_ repeats. The BLASTN search of the flanking regions did not yield any significant hits in the *An. nili *genome.

**Table 1:** Primers designed for the microsatellite loci using the *An. nili *genome sequence.

Locus	Forward primer	Reverse primer	Size of PCR product
B115	CACGCTGGCTAAGAGGAAAC	CTGGTCTCTAGCACCCGAAG	559 bp
2C157	GAGAGCGGCTGTTCGTAATC	CGGACTGGCGAAATAAACAT	506 bp
A154	CACAGGGACGCCTAAAACAT	GACCCGCGTAACTAGGGAAT	537 bp

### Locations of microsatellite markers on the chromosomal map of *Anopheles nili*

**Figure 1:**
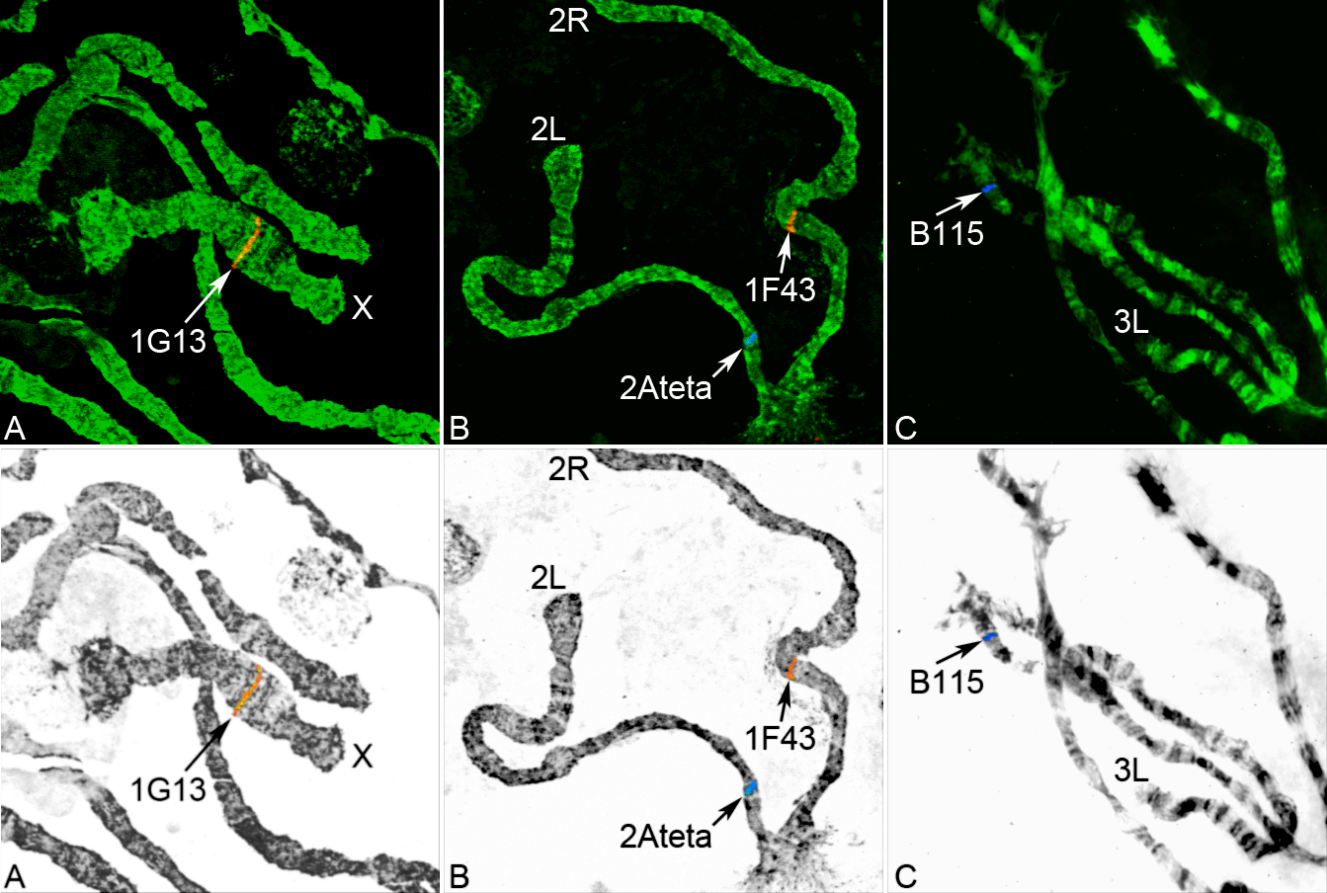
**FISH of microsatellites performed on the *An. nili *chromosomes**. Hybridizations of microsatellite markers 1G13 (A), 2Ateta and 1F43 (B), and B115 (C) with polytene chromosomes are shown. Chromosomes were counterstained with the fluorophore YOYO-1 and hybridized with fluorescently labeled probes Cy5 (blue) and Cy3 (red). The top panel shows fluorescent images of chromosomes after FISH. The bottom panel shows inverted grayscale chromosome images with color labels and distinct banding patterns.

**Figure 2:**
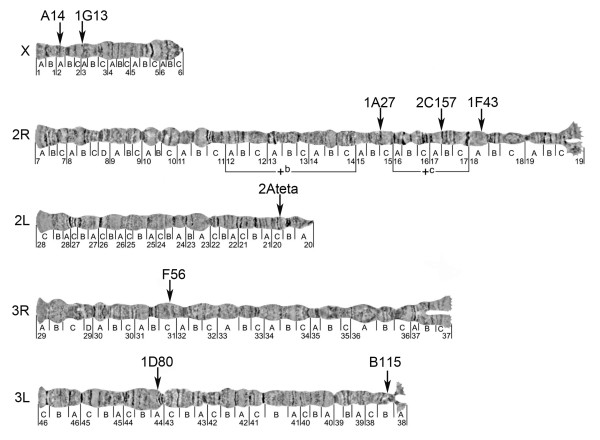
**Physical chromosome map of the *An. nili *microsatellites**. Chromosomal locations of nine microsatellite markers on polytene chromosomes are shown by arrows. Two polymorphic chromosomal inversions are indicated by brackets.

The *An. nili *chromosomal complement in ovarian nurse cells consists of five chromosomal arms: X, 2R, 2L, 3R and 3L. All nine microsatellites were mapped to unique locations on all autosomes and the X chromosome using FISH (Figure 1). We assigned these microsatellites to the precise positions on the recently developed polytene chromosome map of *An. nili *(Figure 2, Table 2). Two microsatellites hybridized to the X chromosome in subdivisions 2A and 3A; three microsatellites localized to the 2R arm in subdivisions 15C, 17AB and 18A; two microsatellites were mapped to the 3L arm in regions 38B and 44A; and arms 2L and 3R each hybridized with only one microsatellite marker in sections 20C and 31C, respectively. Only one microsatellite, 2C157, was mapped inside the previously described polymorphic inversion *2Rc*. Microsatellite 1A27 localized to subdivision 15C located between inversions *2Rb *and *2Rc*. Microsatellite 1F43 was mapped to subdivision 18A located next to the proximal breakpoint of inversion *2Rc*.

**Table 2:** Location of the *An. nili *microsatellite markers on polytene chromosomes.

	Locus	Accession number ^a^ (with length)	Location on the *An. nili *map	Location on the *An. gambiae *map	Best e-value for BLASTN
1.	A14	AJ536274 (467 bp)	X: 2A	X: 3D	3e-08
2.	1G13	AJ536271 (441 bp)	X: 3A	-	-
3.	1A27	AJ536268 (300 bp)	2R: 15C	-	-
4.	2C157	AJ536273 (404 bp) JF742788 (2,967 bp)	2R: 17AB	2R: 10A	7e-04
5.	1F43	AJ536270 (462 bp)	2R: 18A	-	-
6.	F41	AJ536277 (423 bp)	2R: *unknown *^*b*^	2R: 16D	2e-27
7.	2Ateta	AJ536272 (479 bp)	2L: 20C	3L: 45A	3e-17
8.	F56	AJ536278 (474 bp)	3R: 31C	-	-
9.	A154	JF742789 (1,522 bp)	3R: *unknown *^*b*^	3R: 30E	6e-43
10.	B115	AJ536276 (350 bp) JF742787 (10,057 bp)	3L: 38B	2L: 20D	3e-24
11.	1D80	AJ536269 (483 bp)	3L: 44A	-	-

^*a*^ AJ refers to clone sequences; JF refers to assembled sequences.

^*b*^ Arms determined based on the chromosome homology established earlier [19].

"-" no homologous sequence found in the *An. gambiae *genome using BLASTN.

### Microsatellite mapping through comparative genomics with *Anopheles gambiae*

In this study, we identified sequences in the *An. gambiae *genome that are homologous to six microsatellite loci of *An. nili *(Table 2). The remaining five loci did not have significantly similar sequences in the *An. gambiae *genome. Markers A14, 2C157, 2Ateta, and B115, which we mapped in *An. nili *by FISH, were placed to specific regions of homologous chromosome arms in *An. gambiae *by BLASTN. The BLASTN results confirmed arm homologies between the two species that we determined in our previous study [32]. In addition, we mapped *in silico *microsatellites A154 and F41, which were not previously mapped by FISH. We used the Illumina-based genome sequence assembly of *An. nili *for A154 and the clone sequence for F41 to perform BLASTN against the *An. gambiae *genome. According to the established arm homology, we assigned microsatellites A154 and F41 to 3R and 2R chromosome arms of *An. nili*, respectively (Table 2).

### Inversion polymorphism in *Anopheles nili*

To test if inversions *2Rb *and *2Rc *are in LD, we karyotyped 44 *An. nili *females collected in Dinderesso, Burkina Faso. Inversion frequencies were calculated jointly for these individuals and for 56 previously karyotyped females from the same village [32]. We found a highly significant LD between the two inversions (P = 0.00054), i.e., these inversions occur together much more often than expected. Frequencies of inverted and standard *2Rb *variants were almost equal (0.51 and 0.49 for the standard and inverted arrangement, respectively). However, a highly significant departure from Hardy-Weinberg proportions due to a deficit in heterozygotes (e.g., positive *F*_*IS*_ value) was observed at this locus (*F*_*IS*_ = +0.603, P < 0.0001 single test level). Inversion *2Rc *occurred at high frequency in the sample (0.825), with no significant deviation from Hardy-Weinberg equilibrium (HWE) (P = 0.49) (Table 3).

**Table 3:** Chromosome inversion polymorphism in the *An. nili *population from Dinderesso, Burkina Faso.

	*2Rb*	*2Rc*
Inversion frequency	0.490	0.825
*F*_*IS*_^a^	+ 0.603	+ 0.070
P (HW)^b^	<0.0001	0.49

^a^*F*_*IS*_ was computed as in [59].

^b^ Exact test for conformance to Hardy-Weinberg equilibrium, as implemented in the software in GENEPOP V4.0 [58].

## Discussion

Availability of readable polytene chromosomes in *An. nili *allowed us to develop a map of microsatellite markers and to study polymorphism of chromosomal inversions. Among the three experimental approaches used to map microsatellite markers to chromosomes, using cloned inserts and genome sequence assembly of *An. nili *to amplify and hybridize microsatellites was more successful than using microsatellite fragments amplified with primers for population genetics studies [1,27] (Figure 1). Larger DNA fragments were more suitable for effective labeling by the random primer method than smaller fragments obtained with primers for population genetic studies [27]. In addition to these experimental approaches, we conducted BLASTN searches of the *An. nili *genome fragments with microsatellites (both Illumina generated and cloned) against the *An. gambiae *genome to assign microsatellite loci to chromosome arms according to the synteny between *An. nili *and *An. gambiae *[32] (Table 2). Although, X, 2R, and 3R are homologous between the two species, the 2L arm of *An. gambiae *corresponds to the 3L arm of *An. nili, *and the 3L arm of *An. gambiae *corresponds to the 2L arm of *An. nili, *indicating the presence of a whole-arm translocation. Because of the high number of inversions fixed between the two species, the genome of *An. gambiae *cannot be used as a reference for precise positioning of microsatellites on the *An. nili *chromosomes. In our previous study, we calculated the minimum number of fixed inversions among *An. nili*, *An. gambiae*, and *An. stephensi *and concluded that *An. nili *is, at least, as diverged from *An. gambiae *as *An. stephensi *[32]. In addition to the fixed inversion differences, *An. nili *has a distinct pattern of polymorphic inversions. Therefore, the chromosomal positions of homologous loci with respect to polymorphic inversions will be different in the two species.

The developed microsatellite map (Figure 2) improved our understanding of the population genetic structure of *An. nili. *A recent study using 11 microsatellite markers demonstrated significant genetic differentiation of the *An. nili *population of Kenge in the DRC as compared to the *An. nili *populations in Central and West Africa [1]. Both local adaptation and geographic isolation could cause this differentiation. Extensive allele sharing between populations and homogeneity across loci suggested that enhanced genetic drift rather than selection was responsible for the observed pattern. Although it is unlikely that all loci would be within or close to the same inversion, chromosomal mapping of the markers was needed to determine the degree of their independence. Our study demonstrated that the microsatellite locations are not limited to one or a few specific regions in the genome but spanned the entire five-arm complement (Figure 2). Because most of these markers are physically unlinked, we conclude that enhanced genetic drift, rather than selection was responsible for reduced variability and increased differentiation of the Kenge, DRC population (see also Additional file 1). These data strongly suggest the role of the equatorial forest block as a barrier to gene flow between the south-African and north-African populations of *An. nili*.

Among the mapped microsatellite loci, 1A27 and A14 were found to be in particularly strong and significant departure from HWE due to a deficiency of heterozygotes in West Africa (Burkina Faso and Senegal) but not in Central Africa (Cameroon) [1]. We also detected a highly statistically significant departure from HWE due to a deficit in heterozygotes (*F*_*IS*_ = +0.603, P < 0.0001 single test level, Table 3) at inversion *2Rb *in the village of Dinderesso in Burkina Faso among 100 karyotyped females. It is possible that the *2Rb *inversion plays a role in local adaptation and subdivides *An. nili *into populations with limited gene flow. This process or the presence of null alleles could cause heterozygote deficiency at microsatellite loci. In contrast, inversion *2Rc *demonstrated no significant deviation from HWE (Table 3). However, we found a highly significant LD between the two inversions (P = 0.00054). Microsatellite 1A27 is located between *2Rb *and *2Rc *and it could be affected by the LD and reduced recombination in the vicinity of chromosomal breakpoints (Figure 2). Future studies should determine whether this LD is caused by physical linkage or selection. 2C157 is the only microsatellite located inside an inversion; and it does not demonstrate deficiency of heterozygotes. This locus is in the middle of inversion *2Rc *where recombination could be close to normal. Moreover, significant departure from HWE due to a deficiency of heterozygotes was demonstrated for inversion *2Rb *but not for *2Rc. *Marker A14 is located on the X chromosome, which lacks polymorphic inversions, suggesting that genetic differentiation is not limited to the inversions (see Additional file 1 for locus-specific *F*_*ST*_ estimates). Microsatellites in Hardy-Weinberg disequilibrium could also be associated with genes responsible for epidemiologically important ecological adaptations. Indeed, the microsatellite motif of A14 is located 259 bp upstream from the start codon of an open reading frame in the *An. nili *genome, and the sequence homologous to the A14 clone is found in the 5'UTR and the first exon of the *An. gambiae *gene AGAP000275. According to gene ontology annotation, the protein encoded by this gene has oxidoreductase activity. The transcript of AGAP000275 has demonstrated significant differential expression in a variety of mosquito tissues and life stages. Significant differences have been shown between: different stages of embryonic development, between embryonic serosa and embryo [41], between blood-fed and non-blood-fed females, between fat body and ovaries, between males and females, between adults and larva [42], between hemolymph and carcass [43], between West and East African strains of S form gravid females [44], between larval anterior midgut and hindgut [45], between larval salivary gland and whole organism [46]. Significant 1.2-fold increase in the transcription level of AGAP000275 has also been found between females 6 hours and 24 hours after mating [47]. Altogether, these data suggest strong selection acting on AGAP000275 in *An. gambiae *that might translate into non-neutral polymorphism distribution at locus A14 in *An. nili*. Sequences homologous to other *An. nili *microsatellite loci with significant BLASTN hits in the *An. gambiae *genome were found outside genes, except microsatellite B115, which was located within the second intron of gene AGAP004824.

Our recent mapping of 12 microsatellites to *An. stephensi *chromosomes has demonstrated that the chromosomal position of microsatellites may affect estimates of population genetic parameters [48]. In a similar study of *An. funestus*, 16 microsatellites were physically mapped to polytene chromosomes, and the location of microsatellites based on the inversions were determined [49]. Interestingly, microsatellites located between inversions *3Ra *and *3Rb *in *An. funestus *were found in LD with these inversions in Burkina Faso [50] but not in Cameroon [30], reflecting different evolutionary outcomes in different eco-geographic regions. Altogether, these studies point to the importance of physical mapping of molecular markers exposed to contrasted evolutionary dynamics for unravelling the demographic and evolutionary history of malaria vectors. This paper provides the necessary toolbox for such endeavour to be pursued in *An. nili*.

## Conclusions

Our study demonstrates that the chromosomal position of microsatellites is informative for interpretation of population genetics data and highlights the importance of developing physical maps for nonmodel organisms. Next-generation sequencing can be used for designing microsatellite primers to obtain longer microsatellite-containing probes and improve FISH mapping. An Illumina-based genome sequence assembly can also be used for identifying homologous loci in the reference genomes and assigning microsatellite markers to chromosomal arms in a species of interest based on synteny. The integrated chromosomal map of microsatellites and inversions will allow for more complete characterization of *An. nili *in future population genetics studies. It will be possible to test for a LD among and between inversions and microsatellites, genetic differentiation at microsatellite loci located inside and outside inversions, and genetic differentiation according to the distance from inversion breakpoints. In addition, the new genetic map could be used for designing quantitative trait loci mapping studies for this species.

## Methods

### Wild mosquito collection, preservation, and species identification

*Anopheles nili *adult females were collected by pyrethrum spraying and bednet traps in the village of Dinderesso (11°14'N; 4°23'W) in Burkina Faso. *Anopheles nili *larvae were collected in a river in Dinderesso, Burkina Faso. Specimens were identified in the field as members of the *An. nili *group by using morphological identification keys [51-53] and were further characterized by molecular assays as *An. nili s.s. *[54]. Females were dissected under a microscope, and their ovaries at the appropriate stage were preserved in Carnoy's fixative solution (3 parts of ethanol: 1 part of glacial acetic acid by volume). Ovaries were kept at room temperature overnight before being stored at -20°C. Larvae were preserved in Carnoy's fixative solution and stored at -20°C.

### Genome sequencing and BLASTN

The genome assembly for *An. nili *was obtained by sequencing of genomic DNA isolated from two larvae collected in Dinderesso, Burkina Faso. Genomic DNA was isolated using the Qiagen DNeasy Blood and Tissue Kit (Qiagen Science, Germantown, MD, USA). The library preparation and sequencing was performed on the Illumina Genome Analyzer IIx, using 72 bp paired-end processing at Ambry Genetics Corp. (Aliso Viejo, CA, USA). Samples were prepared using the Illumina protocol outlined in "Preparing Samples for Sequencing Genomic DNA" (Part # 11251892 Rev. A 2007). Briefly, DNA fragment ends were repaired and phosphorylated using Klenow, T4 DNA Polymerase and T4 Polynucleotide Kinase. Next, an 'A' base was added to the 3' end of the blunted fragments, followed by ligation of Illumina paired-end adaptor via T-A mediated ligation. The ligated products were size selected by gel purification and then PCR amplified using Illumina Paired-End primers. The library size and concentration were determined using an Agilent Bioanalyzer. The library was seeded onto the flowcell at 8 pM, yielding approximately 275 K clusters per tile, and it was sequenced using 73 cycles of chemistry and imaging (73 cycles) for read 1 and read 2. Initial data processing, including extraction of cluster intensities and base calling, was done using RTA 1.6.47 (SCS version 2.6.26). Sequence quality filtering scripts were executed in the Illumina CASAVA software (ver 1.6.0, Illumina, Hayward, CA). Quality metric data included the approximate proportion of sequences with 1, 2, 3 or 4 errors, IVC plots, and visualizations of cluster intensity over the duration of the sequencing run. The BLASTN algorithm was used to identify homologous sequences in the *An. gambiae *genome, which is available at VectorBase [55]. The BLASTN algorithm was also used to find larger genomic fragments with microsatellite loci in the *An. nili *genome using a server and the Geneious 5.1.5 software http://www.geneious.com, a bioinformatics desktop software package produced by Biomatters Ltd http://www.biomatters.com.

### Probe preparation

Three approaches were utilized for the microsatellite probe preparation. First, microsatellites were directly amplified from the *An. nili *genomic DNA using previously designed primers [27]. Approximately 90-230 bp-long fragments were amplified. Second, plasmid clones with microsatellites were used as templates for insert amplification. In this case, 300-483 bp-long fragments were amplified from the pUC18 plasmid DNA using standard M13 forward and reverse primers (Fermentas, Inc., Glen Burnie, MD, USA). Third, primers were designed for three microsatellites using the Primer3 program [56] based on sequences identified by BLASTN in the genome assembly of *An. nili *(accession numbers: JF742787, JF742788, JF742789). The size of these fragments was about 506-559 bp. PCR conditions were as follows: 94°C for 5 min; 45 cycles of 94°C for 45 s, 50°C for 45 s and 72°C for 30 s; and 72°C for 5 min. DNA was purified using the GE healthcare illustra GFX PCR DNA and Gel Band Purification Kit (GE Healthcare UK Ltd, Buckinghamshire, UK). Probes were labeled using Cy3-AP3-dUTP or Cy5-AP3-dUTP (GE Healthcare UK Ltd., Buckinghmashire, UK) fluorophores by a Random Primer DNA Labelling System (Invitrogen Corporation, Carlsbad, CA, USA).

### Chromosome preparation and FISH

To obtain chromosomal slides, follicles of ovaries were separated in 50% propionic acid. Then a cover slip was used to squash the follicles. The quality of slides and the banding pattern of polytene chromosomes were analyzed using an Olympus CX-41 phase contrast microscope (Olympus America Inc., Melville, NY, USA). Slides then were dipped into liquid nitrogen, cover slips were removed, and slides were dehydrated in 50%, 70%, 90% and 100% ethanol. Slides were air dried and used for further experiments. Labelled probes were hybridized at 42°C to *An. nili *polytene chromosome slides overnight. Then, slides were washed in 0.2 X SSC (Saline Sodium citrate, 0.03 M sodium chloride and 0.03 M sodium citrate) at 42°C and room temperature. Chromosomes were stained using YOYO-1 (Invitrogen Corporation, Carlsbad, CA, USA), and slides were mounted in 1,4-diazabicyclo[2.2.2]octane (DABCO) antifade solution. A Zeiss LSM 510 Laser Scanning Microscope (Carl Zeiss MicroImaging, Inc., Thornwood, NY, USA) was used to detect fluorescent signals. Microscopic images were taken from the signal, and the locations of signals were determined using a standard cytogenetic photo map of *An. nili *[32].

### Image processing

Confocal images were processed using ImageJ and Adobe Photoshop software as described elsewhere [57]. Briefly, color channels were split from the initial RGB image into separate images. Each channel image was converted into the monochrome image by using a 'Channel mixer' and then inverted. The inverted monochrome image was adjusted by using a 'Curves' tool until the background is removed and each chromosome of the spread becomes fuzzy-edged. The reduction of noise was achieved by blurring of each pixel with the Gaussian blur filter tool. The quality of the image was improved by additional application of the 'Curves' and/or subtraction of the 'Relative white'. Finally, green channel image with chromosomes was merged with monochrome image FISH signals. Processing yielded contrasted, inverted, grayscale images with color labels, which are more suitable for mapping.

### Population genetics analyses

Homozygous and heterozygous inversions were scored using the chromosomal map published earlier [32]. Alternative chromosomal arrangements were considered as different alleles of the same locus, and conformance to Hardy-Weinberg equilibrium was tested with Fisher's exact tests available in GENEPOP V4.0 [58]. A *F*_*IS*_ value was computed as in [59]. LD between the inversions *2Rb *and *2Rc *was assessed using the log likelihood ratio statistic (*G*-test) available in GENEPOP V4.0 [58].

## List of abbreviations

BLASTN: Basic Local Alignment Search Tool for Nucleotide sequences; DABCO: 1,4-diazabicyclo [2.2.2] octane; FISH: fluorescent *in situ *hybridization; HWE: Hardy-Weinberg equilibrium; LD: linkage disequilibrium.

## Competing interests

The authors declare that they have no competing interests.

## Authors' contributions

IVS designed research; AP, IVS, and MVS performed karyotyping and physical mapping; CAN and CN conducted field work and mosquito identification; FS, MVS, and IVS analyzed data; MW prepared plasmid DNA; AP and IVS prepared genomic DNA for sequencing; IVS performed BLASTN searches using VectorBase and a Geneious software; AP and IVS wrote the paper, which was critically revised by CAN, FS, and MVS. All authors read and approved the final manuscript.

## Supplementary Material

Additional file 1 **Re-analysing genetic differentiation between *Anopheles nili *populations from West and Central Africa**. The file contains the genotypic data re-analyzed according to microsatellite loci cytological location. Locus-specific *F*_*ST*_ values are shown in Table S1, together with *F*_*ST*_ estimates across each chromosomal arm and overall. Locus-specific jackknifed mean *F*_*ST*_ estimates (+/- standard deviation) between *An. nili *populations from West and Central Africa are shown in Figure S1. Click here for file
