# Supplementary material for: Improving the population genetics toolbox for the study of the African malaria vector Anopheles nili: microsatellite mapping to chromosomes
Source: Parasit Vectors. 2011 Oct 19;4:202. doi: 10.1186/1756-3305-4-202 (PMC3222614; doi:10.1186/1756-3305-4-202)
Supplement: Additional file 1 — Re-analysing genetic differentiation between Anopheles nili populations from West and Central Africa. The file contains the genotypic data re-analyzed according to microsatellite loci cytological location. Locus-specific FST values are shown in Table S1, together with FST estimates across each chromosomal arm and overall. Locus-specific jackknifed mean FST estimates (+/- standard deviation) between An. nili populations from West and Central Africa are shown in Figure S1. [file 1756-3305-4-202-S1.DOC]

**Additional file 1**

**Re-analysing genetic differentiation between *Anopheles nili* populations from West and Central Africa.**

We re-analyzed the genotypic data from Ndo *et al.* (2010) [1] according to microsatellite loci cytological location using Genepop V4.0 [2]. Locus-specific *F_st_* values are shown in Table S1, together with *F_st_* estimates across each chromosomal arm and overall. *F_st_* estimates were computed as in Weir and Cockerham (1984) [3]. Statistical significance was assessed using an unbiased estimate of the P-value of a log-likelihood (G) based exact test performed on genotypic data [4], available in Genepop V4.

Data were analyzed according to three schemes:

1. Kenge (Democratic Republic of Congo) population vs all other populations (*e.g.*, genetic cluster 1 vs cluster 2 identified by Ndo *et al.* 2010 [1]) ;
2. all populations (one sample from Senegal, Burkina Faso, Côte d’Ivoire, Nigeria, DRC, respectively and four samples from Cameron) ;
3. only those populations from Burkina Faso and Cameroon where the present study showed chromosomal inversions *2Rb* and *2Rc* are polymorphic (five samples).

All microsatellite loci, on all chromosomes detected significant levels of genetic differentiation among the sampled *An. nili* populations, with single-locus *F_st_* ranging from 0.034 to 0.228 in the cluster analysis (e.g. when the genetically divergent Kenge population is compared to all other West and Central African *An. nili* populations pooled, see [1]), and from 0.003 to 0.063 in the overall analysis. Higher levels of genetic differentiation were generally observed with loci on chromosomal arms 2L and 3R, whereas the lowest *F_st_* estimates were observed with loci on 2R, where polymorphic inversions are known to occur. When only populations from Burkina Faso (one sample) and Cameroon (four samples) are compared, no significant genetic differentiation is observed at microsatellites loci located on the X chromosome, whereas autosomal loci showed a more contrasted pattern of differentiation, with differentiation hot spots on chromosomal arms 2R and 3L.

To further explore variation in locus-specific *F_st_* estimates between populations, we calculated jackknifed means of *F_st_* for each locus across all populations in the dataset (excluding one population at a time and calculating locus-specific *F_st_* estimates across the remaining populations) for datasets with more than two populations (e.g. excluding cluster analysis where only two samples were compared) (Figure S1). Calculations were conducted with FSTAT v2.9.4 software [5]. Loci on chromosomal arm 2R consistently showed low levels of differentiation between populations in the global dataset, irrespective of the population being dropped out of the analysis, whereas loci on other chromosomes demonstrated much higher levels of variation. This suggests that differentiation signal on 2R is quite robust to geographical variation in the sampled *An. nili* populations. When only the Cameroon and Burkina Faso *An. nili* populations are compared, loci on 2R consistently showed significant genetic differentiation between populations, with some variation according to locus position on the chromosomal arm. In particular, locus 2C157, mapped within inversion *2Rc*, showed the highest variance in *F_st_* estimates. This finding might reflect differences between populations in the inversion frequency. However, in the absence of karyotype data, any attempt to link chromosomal and microsatellite polymorphism and divergence within as well as between natural populations is not productive and would result, at best, in weak inference.

**References**

**Table S1 - Locus-specific *F_st_* estimates between *An. nili* populations from West and Central Africa.**

*Arms determined based on chromosome homology [6].

Note that locus 2C157 maps within polymorphic inversion *2Rc.* All other loci are located outside known chromosomal inversions.

The karyotype of specimens included in this study is unknown.

**Figure S1 - Locus-specific jackknifed mean *F_st_* estimates (+/- standard deviation) between *An. nili* populations from West and Central Africa.** A) Complete dataset with nine geographical populations sampled throughout West and Central Africa; B) Cameroon and Burkina populations only (five samples) [1].
